# Plasma protein profiling in patients undergoing coronary artery bypass grafting surgery and clinical significance

**DOI:** 10.18632/oncotarget.16366

**Published:** 2017-03-18

**Authors:** Zhi-Peng Guo, Hai-Tao Hou, Rui Jing, Zhen-Guo Song, Xiao-Cheng Liu, Guo-Wei He

**Affiliations:** ^1^ Center for Basic Medical Research, TEDA International Cardiovascular Hospital, Chinese Academy of Medical Sciences & Peking Union Medical College, Beijing Shi, China; ^2^ Department of Cardiovascular Surgery, TEDA International Cardiovascular Hospital, Chinese Academy of Medical Sciences & Peking Union Medical College, Beijing Shi, China; ^3^ Department of Cardiology, TEDA International Cardiovascular Hospital, Chinese Academy of Medical Sciences & Peking Union Medical College, Beijing Shi, China; ^4^ The Heart Center, The Affiliated Hospital of Hangzhou Normal University, Hangzhou, China; ^5^ Medical College, Zhejiang University, Zhejiang, China; ^6^ Department of Surgery, Oregon Health & Science University, Portland, OR, USA

**Keywords:** coronary artery disease, coronary artery bypass grafting, platelet factor 4, secreted frizzled-related protein 1

## Abstract

This study was designed to identify the protein profiling in patients with triple vessel coronary artery disease (CAD) undergoing CABG, in order to detect CAD-related differential proteins in these patients. CABG patients with triple vessel disease with/without left main stenosis (*n* =160) were compared to normal coronary angiographic subjects (*n* =160). Plasma samples of 20 males and 20 females in each group were analyzed with iTRAQ technique. ELISA test was used to test the chosen proteins from iTRAQ results in plasma samples from a new cohort of the CABG group (*n*=120, male/femal=61/59) and control (*n* =120, male/female=60/60). iTRAQ detected 544 proteins with 35 up-regulated and 41 down-regulated (change fold > 1.2 or < 0.83, *p* < 0.05). Three proteins including platelet factor 4 (PF4), coagulation factor XIII B chain (F13B), and secreted frizzled-related protein 1 (sFRP1) were selected for validation by using ELISA that demonstrated significant up-regulation of PF4 and sFRP1 (*p* < 0.05). There was a positive correlation between these proteins and CAD (*p* < 0.05) and myocardial infarction history (*p* < 0.05). Thus, we for the first time have found 76 proteins differentially expressed in plasma of CABG patients. The thrombotic disease/inflammation progress-related protein PF4 and sFRP1, a member of the Wnt/fz signal-transduction pathway and related to myocardial repair, are significantly up-regulated in triple-vessel disease with/without left main stenosis. PF4 may be developed as a biomarker for the diagnosis of the severity of CAD requiring CABG procedure.

## INTRODUCTION

Cardiovascular disease is the leading cause of death all over the world. The exact mechanism of CAD remains unclear. Regarding the molecular mechanism of the development of CAD, more than 100 single nucleotide variants (SNVs) associated with CAD have been confirmed by genome wide association study [1]. In addition, changes of plasma proteins have been reported to be correlated with the pathological process of CAD such as inflammation, platelet activation and coagulation [2] and more proteins on CAD have been discovered with proteomic technologies [3, 4]. However, despite of the fact that around 800,000 CAD patients undergo coronary artery bypass grafting (CABG) worldwide annually, the protein profiling of the CABG patients have not been reported.

Plasma is the ideal source for proteome analysis due to the fact that it is easily sampled from patients and reflects biological processes. In the human plasma, there are more than 10,000 different proteins that are secreted or shed by cells during different physiologic or pathologic processes [5]. Based on our previous experiences in plasma proteomic studies [6–9], the present study was designed to identify the protein profiling in triple vessel CAD patients undergoing CABG, in order to detect CAD-related differential proteins in CABG patients by determining the correlations between the specific proteins in the specific disease. The results from the present study may help understanding the pathological processes at the protein level and developing more precise diagnostic and treatment strategy towards “Precision Medicine” in CABG procedures.

## RESULTS

### General information of patients

General information and clinical characteristics are shown on Tables 1 and 2. There are significant differences of the diabetes mellitus, hypertension and hyperlipidemia prevalence between CABG and Control groups (*p* < 0.05).

**Table 1:** General information of patients in iTRAQ test

	Group				*p*
	CABG Male	CABG Female	Control Male	Control Female	
Age(yrs)*	60.6+8.7	60.8+6.5	58.0+10.9	61.2+6.3	0.590
Height(cm)*	169.0+5.6	159.0+4.8	172.4+6.5	158.4+2.5	0.000
Weight(kg)*	79.7+12.5	64.4+7.5	79.2+10.3	63.7+9.3	0.000
BMI*	27.9+3.9	25.5+2.8	26.6+3.0	25.4+3.7	0.081
DM n (%)**	11(55.0%)	9(45.0%)	3(15.0%)	6(30.0%)	0.047
Hypertension n (%)**	11(55.0%)	15(75.0%)	12(60.0%)	11(55.0%)	0.519
Hyperlipidemia n (%)**	12(60.0%)	15(75.0%)	9(45.0%)	6(30.0%)	0.029
Myocardial Infarction n (%)**	4(20.0%)	3(15.0%)	0	0	0.046

* mean+SD; ** number (%); BMI= Body mass index; DM=diabetes mellitus

**Table 2:** General information in ELISA test

	Group		*p*
	CABG (*n* = 120)	Control (*n* = 120)	
Age(yrs)*	61.1+8.6	58.5+9.1	0.022
Height(cm)*	163.4+7.9	166.0+8.0	0.014
Weight(kg)*	69.0+11.6	70.9+12.9	0.238
BMI*	25.7+3.3	25.6+3.6	0.781
DM n (%)**	48 (40%)	26 (21.7%)	0.002
Hypertension n (%)**	87 (72.5%)	69 (57.5%)	0.011
Hyperlipidemia n (%)**	86 (71.7%)	48 (40%)	0.000
Myocardial Infarction n (%)**	46 (38.3%)	0	0.000

*: present as mean+SD; **: present as number (%); BMI= Body mass index; DM=diabetes mellitus

### iTRAQ analysis and proteins identification

**Figure 1:**
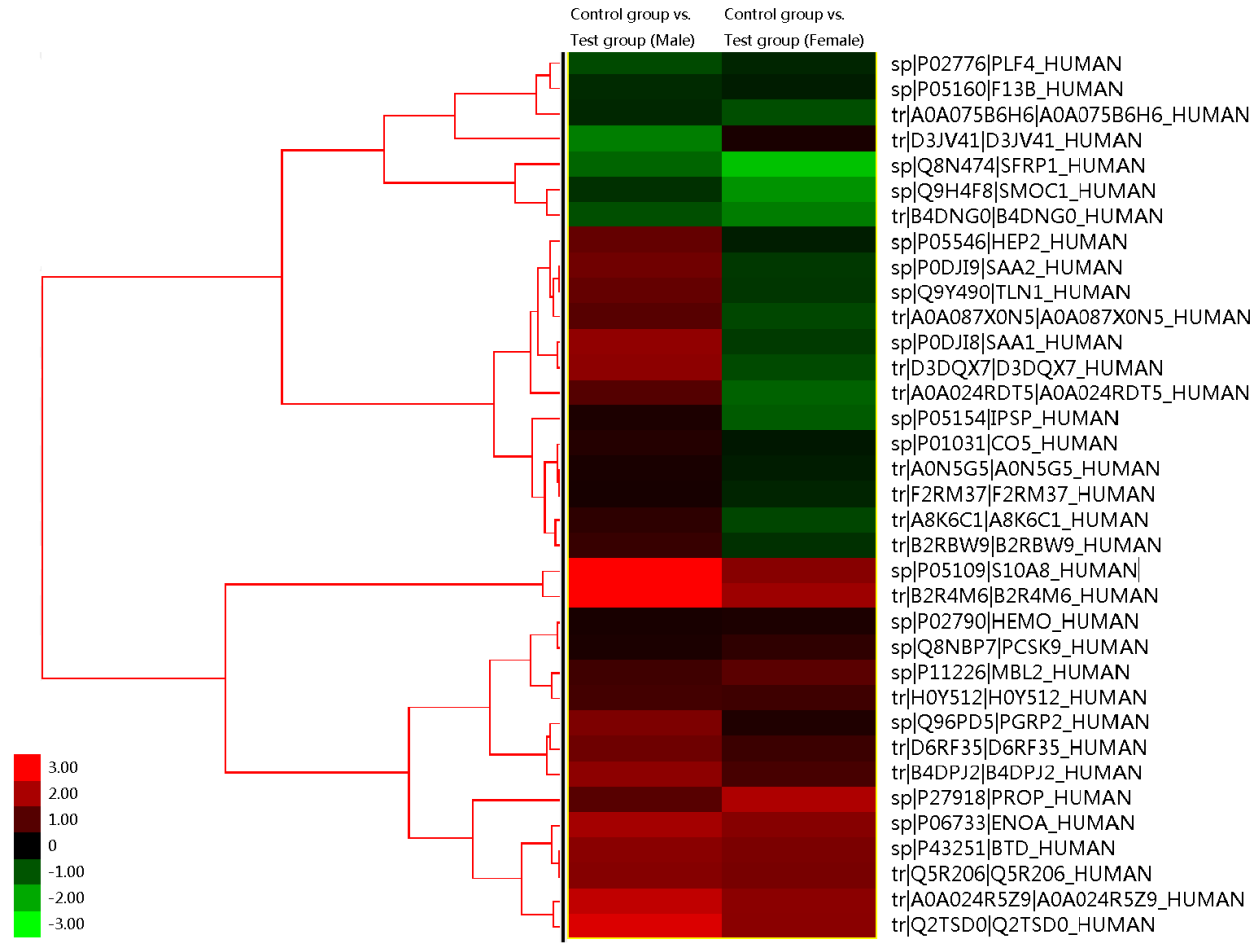
Heatmap from the results of iTRAQ This reflects the protein expression values in different groups and functional cluster analysis of different proteins. The protein expression was compared in male and female, respectively. Proteins with similar functions have a relative shorter Euclidean distance. The categorical annotation bar (left of heatmap) demonstrate the annotation of protein expression values. The right side shows the name of proteins. The red and green colors represent up- and down-regulated proteins, respectively. The numbers in the left lower coner gives the fold of changes of a protein, correlating to the color shown beside the number.

**Figure 2:**
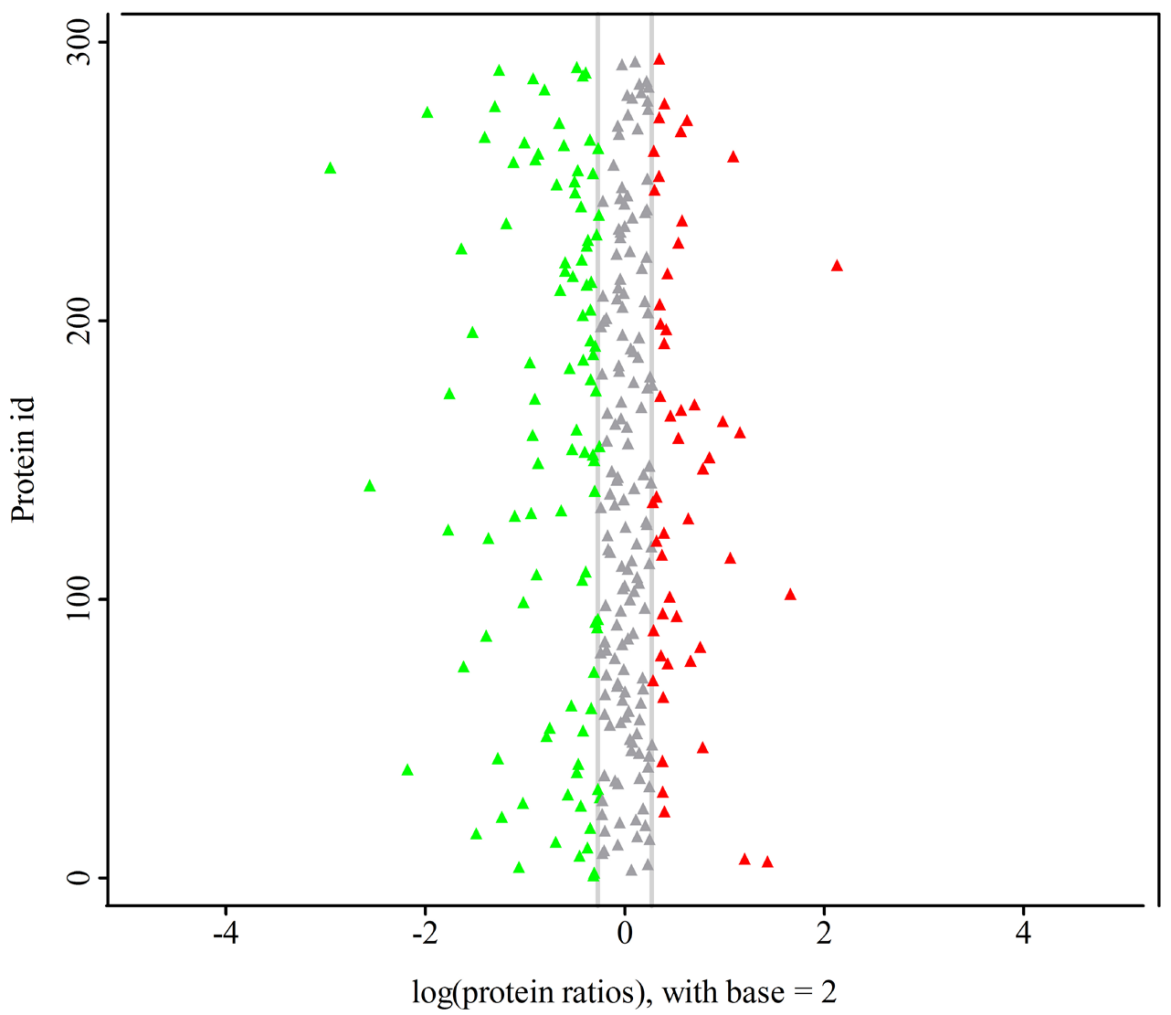
Protein ratio distribution in coronary artery disease, according to the result of iTRAQ X-axis presents differential ratio log-transformed with base 2. All differential proteins including up-regulation (triangles in red) and down-regulation (triangles in green) with the change greater than 1.2 fold compared to the control are included in the figure. However, among all these proteins, only 35 up-regulated and 41 down-regulated differential proteins were statistically significant (CABG vs. control, *p* < 0.05) as mentioned in the text. These proteins are listed in the supplemental Table S1 in details.

**Figure 3:**
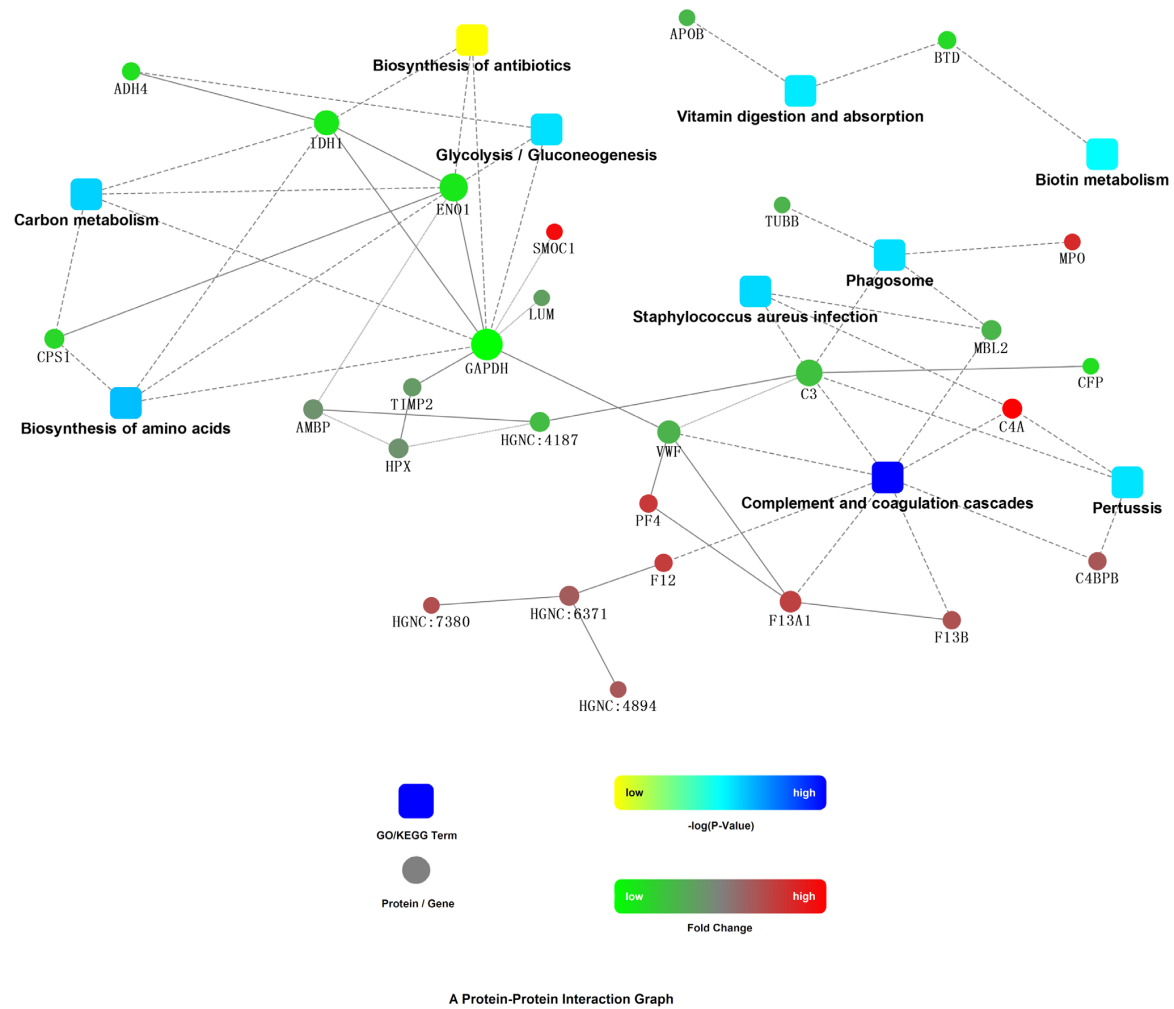
Protein-Protein interaction network STRING, a widely used biological database, was utilized as a search tool for the Retrieval of Interacting Genes/Proteins. The network model is generated with cytoscape web application, based on information gained up to 4 level of functional analysis: fold change of gene/protein, protein-protein interaction, KEGG pathway enrichment and biological process enrichment. Circle nodes showed the identified up- and down-regulated proteins displayed in red and green, respectively. Pathway were colored with gradient color from yellow to blue, yellow for smaller *p*-value, blue for bigger *p*-value. The gray arrows with solid and dashed lines indicated the interaction with bigger and smaller confident score, respectively between the molecules. The Figure suggests that complement and coagulation cascade, biosynthesis of amino acids, exec, are possibly linked with CAD-related protein changes.

iTRAQ identified 544 proteins and 2, 530 unique peptides. Heatmap in Figure 1 reflects the protein expression values in different groups and functional cluster analysis of different proteins. The protein expression was compared in male and female, respectively. Proteins with similar functions have a relative shorter Euclidean distance. The categorical annotation bar (left of heatmap) demonstrate the annotation of protein expression values. The red and green colors represent up- and down-regulated proteins, respectively. Triangles in Figure 2 showed the proteins with ratios greater than 1.2 or less than 0.8. The ratios were log-transformed with base 2. Thirty-five proteins were significantly up-regulated and 41 down-regulated (CABG vs. control, *p* < 0.05). These proteins are shown in Supplementary Table S1. Figure 3 showed the protein-protein interaction graph.

Four proteins in Supplementary Table S1 including coagulation factor XIII B chain (F13B), platelet factor 4 (PF4), secreted frizzled-related protein 1 (sFRP1), and vitamin D-binding protein (DBP) were selected to be validated with ELISA procedure. The selection of these four proteins for validation were based on 1) potential functional significance in CAD; 2) more than 1 peptide was identified by LC-MS/MS; and 3) not been reported before in CAD patients. Among these 4 proteins, F13B, PF4, sFRP1 were up-regulated whereas DBP was down-regulated (*p* < 0.05).

### Validation by ELISA

**Figure 4:**
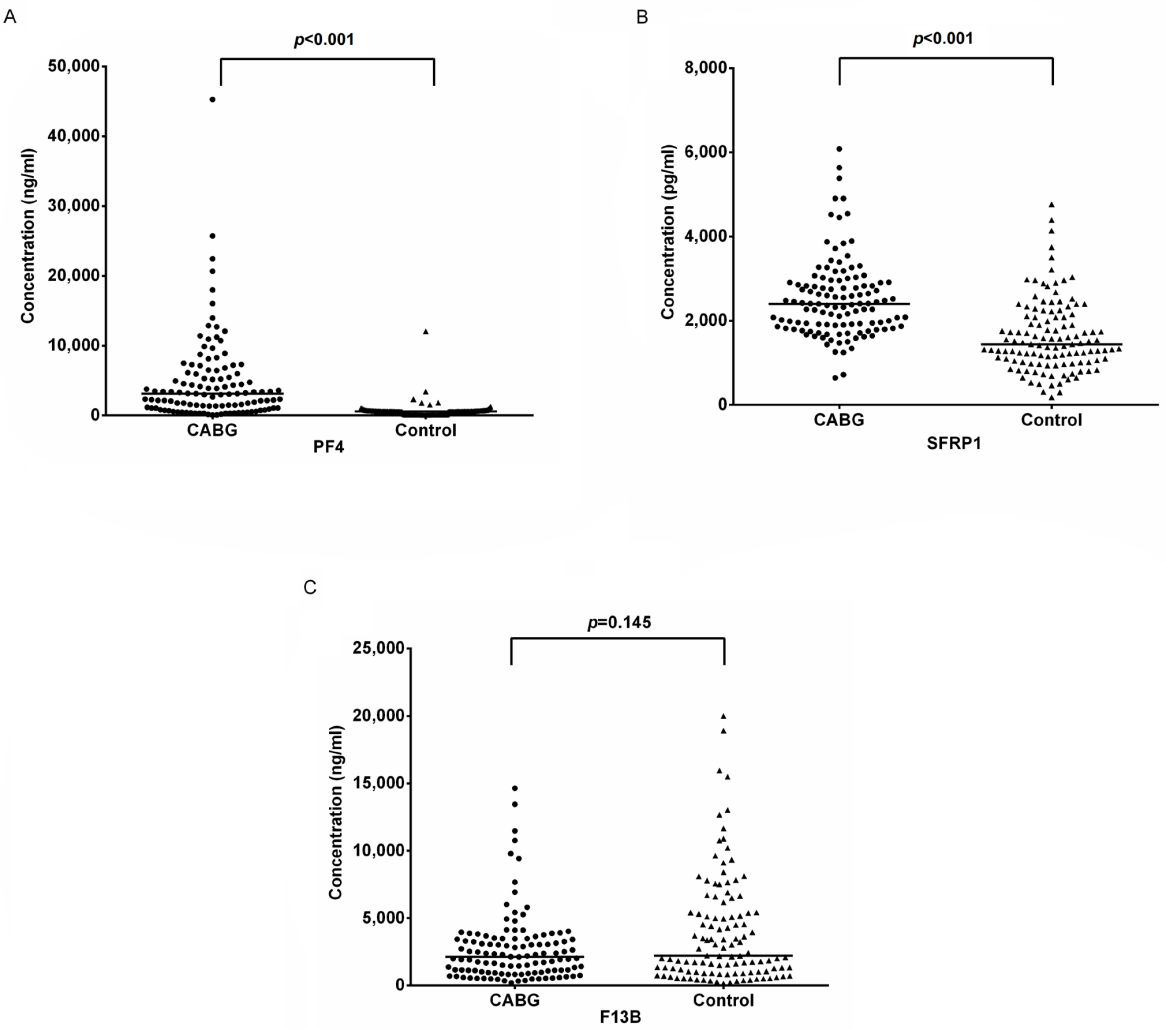
ELISA validation ELISA test for platelet factor 4 (PF4, **A**), secreted frizzled-related protein 1 (sFRP1, **B**), and coagulation factor XIII B chain (F13B) in CABG group (*n* = 160) and control group (*n* = 160). The plasma level of PF4 and sFRP1 were up-regulated significantly (CABG vs. control, *p* < 0.05). However, there were no significant differences of F13B (**C**) between two groups (*p* = 0.145).

In the subsequent ELISA test, the plasma level of DBP, PF4 and sFRP1 were up-regulated significantly (CABG vs. control, *p* < 0.05) (Figure 4). However, there were no significant differences of F13B (C) between two groups (*p* = 0.145). Notably, ELISA did not validate the iTRAQ result of DBP that was down-regulated but it was up-regulated in ELISA.

To rule out the effect of covariates including age, gender, BMI, hypertension, DM, hyperlipidemia, hemoglobin and uric acid, partial correlation coefficient was used for the coefficient analysis between protein and coronary artery disease (Table 3). PF4 and sFRP1 had a positive correlation with CAD diagnosis (*p* < 0.05). Same analysis was performed between protein and myocardial infarction (Table 4). PF4, sFRP1 also had a positive relation with history of myocardial infarction (*p* < 0.05).

**Table 3:** Partial correlation coefficient analysis for the proteins and CAD

	CAD	Age	Gender	BMI	hypertension	DM	hyperlipidemia	Hb	Uric acid	CAD with covariates
PF4	0.629*	0.078	−0.206*	−0.074	0.107	0.077	0.222*	−0.158*	−0.148*	0.621*
sFRP1	0.479*	0.163*	0.030	−0.146*	0.086	0.041	0.189*	−0.015	−0.119*	0.416*
F13B	−0.096	0.184*	−0.237*	−0.179*	−0.100	−0.082	−0.128	−0.146*	−0.225*	−0.066

*: *p* < 0.05; CAD: coronary artery disease, DM: diabetes mellitus, Hb: hemoglobin. Partial correlation coefficient was used for the coefficient analysis between protein and coronary artery disease.

**Table 4:** Partial correlation coefficient analysis for the proteins and MI

	MI	Age	Gender	BMI	hypertension	DM	hyperlipidemia	Hb	Uric acid	MI with covariates
PF4	0.275*	0.078	−0.206*	−0.074	0.107	0.077	0.222*	−0.158*	−0.148*	0.253*
sFRP1	0.276*	0.163*	0.030	−0.146*	0.086	0.041	0.189*	−0.015	−0.119*	0.240*
F13B	−0.025	0.184*	−0.237*	−0.179*	−0.100	−0.082	−0.128	−0.146*	−0.225*	−0.014

*: *p* < 0.05; Partial correlation coefficient was used for the coefficient analysis between protein and coronary artery disease.

**Figure 5:**
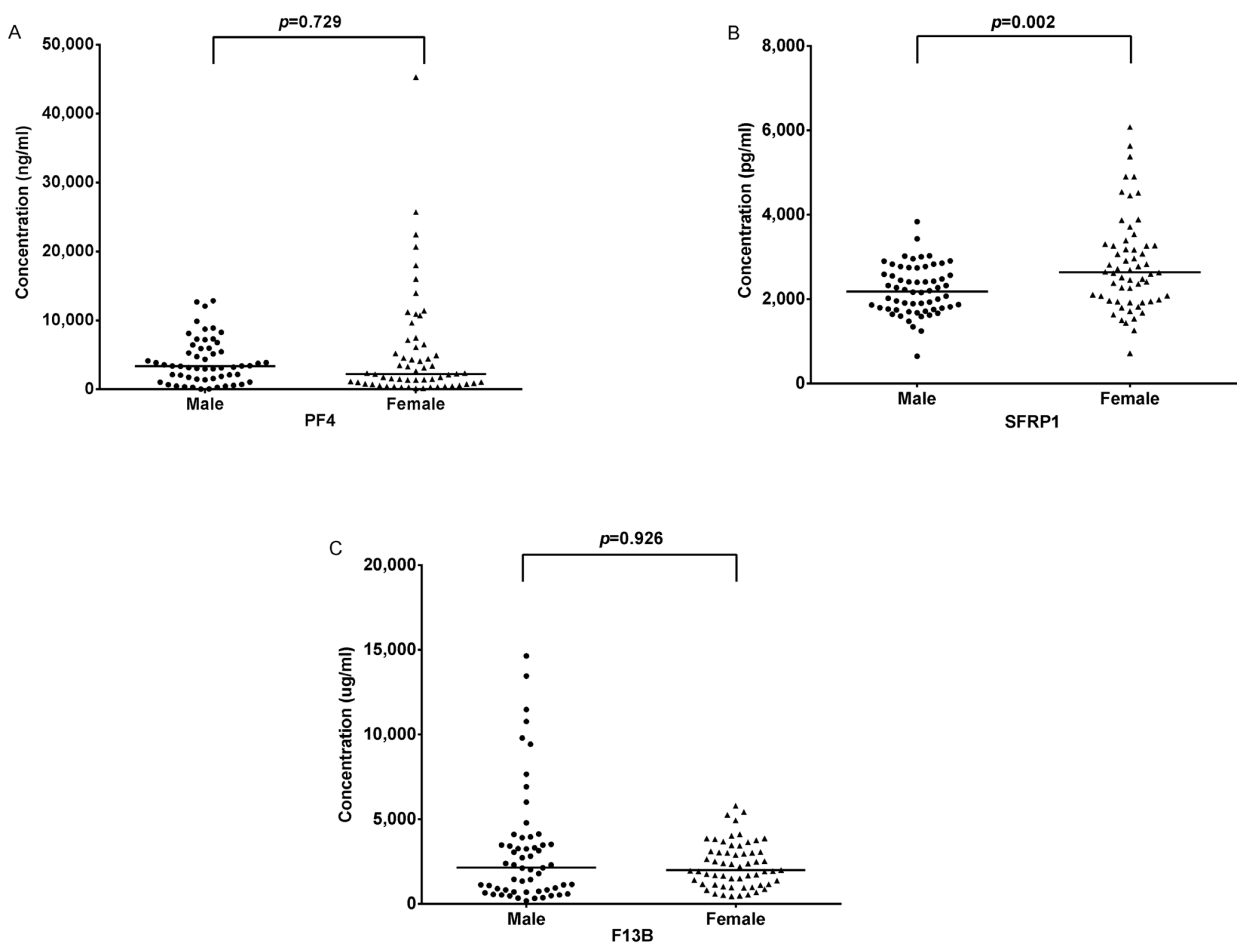
Comparison of plasma levels of differential proteins between males and females There is a significant difference of sFRP1 between two genders, i.e. female patients represented a relatively higher level of sFRP1 (**B**, also see Table 5).

The effect of different genders on proteins in CABG patients was also analyzed. The plasma level of sFRP1 was differently expressed between males and females (*p* < 0.05, Figure 5), i.e. female patients represented a relatively higher level of sFRP1 (*p* < 0.05, Table 5).

**Table 5:** Partial correlation coefficient analysis for the proteins and gender in CAD patients

	Gender	Age	MI	BMI	hypertension	DM	hyperlipidemia	Hb	Uric acid	Gender with covariates
PF4	0.033	−0.171	−0.013	0.281*	−0.090	−0.184*	−0.248*	0.315*	0.357*	0.076
sFRP1	−0.286*	0.134	0.092	−0.185*	0.010	0.045	0.117	−0.021	−0.079	−0.226*
F13B	−0.009	0.108	0.055	−0.058	−0.135	−0.073	−0.234*	0.013	−0.159	−0.047

*: *p* < 0.05; Partial correlation coefficient was used for the coefficient analysis between protein and coronary artery disease.

**Figure 6:**
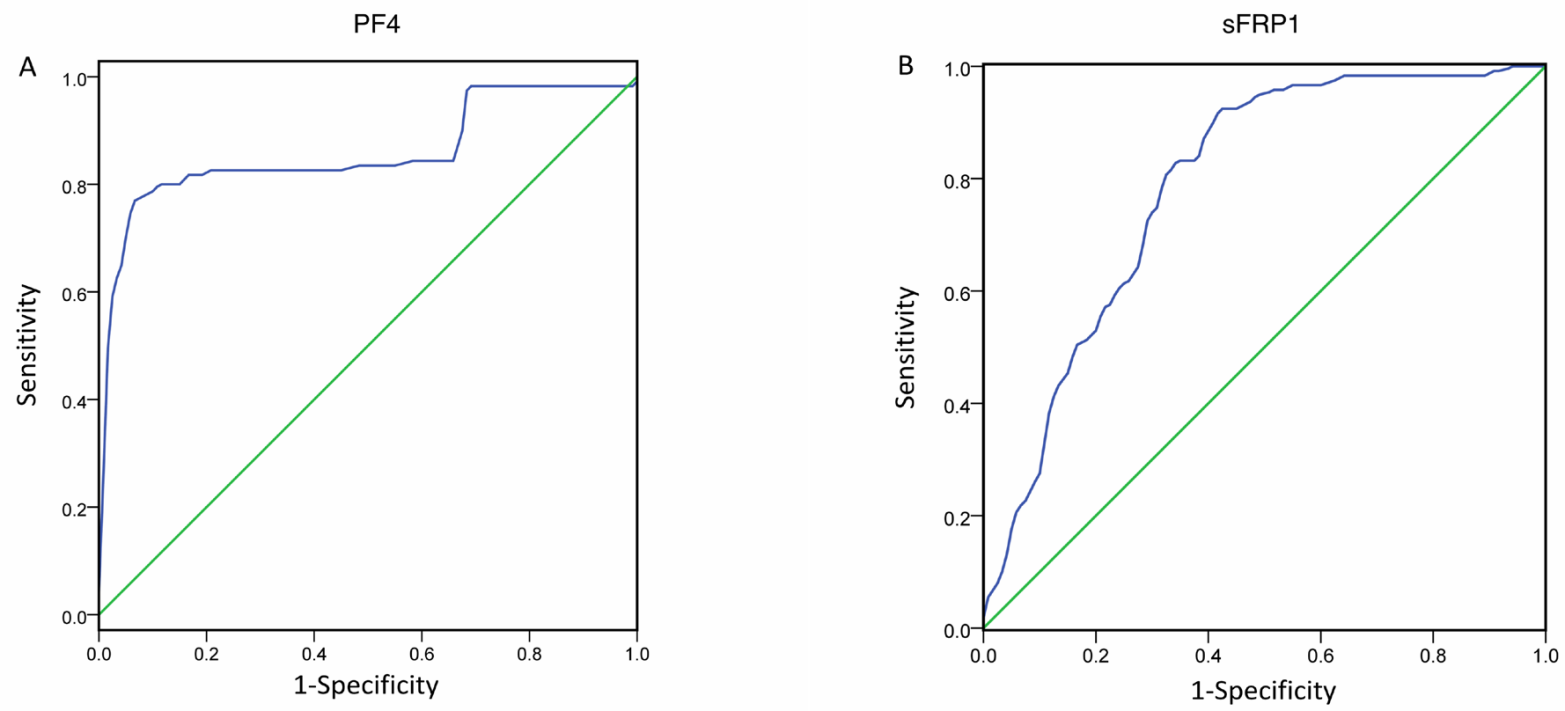
ROC tests ROC tests for PF4 and sFRP1 for the diagnosis of the severity of CAD (*n* = 240 including 120 CABG patients and 120 controls). Areas under the curve are 0.858 and 0.788 for PF4 (**A**) and sFRP1 (**B**), respectively (*p* < 0.05). The best differential point of PF4 was 1021.00 with sensitivity of 77.4% and specificity of 93.3% (YI = 0.707). The best differential point of sFRP1 was 1581.625 (YI = 0.507). The sensitivity and specificity of sFRP1was 92.4%, 58.3%, respectively. ROC curves help selecting optimal differential point for a biomarker or a test during diagnosis.

The ROC tests for PF4 and sFRP1 were shown in Figure 6 (*n* = 240 including 120 CABG patients and 120 controls). Areas under the curve were 0.858 and 0.788 for PF4 (Figure 6 A) and sFRP1 (Figure 6 B), respectively (*p* < 0.05). The best differential point of PF4 was 1021.00 with sensitivity of 77.4% and specificity of 93.3% (YI = 0.707). The best differential point of sFRP1 was 1581.625 (YI = 0.507). The sensitivity and specificity of sFRP1was 92.4%, 58.3%, respectively.

## DISCUSSION

In the present study, we have for the first time found in the plasma of CABG patients that: 1) 35 proteins were up-regulated and 41 were down-regulated; and 2) among the 4 proteins validated in the new group of patients, PF4 and sFRP1 were significantly up-regulated.

In the present study, iTRAQ, an isobaric labeling method, was used as proteomic strategy for detecting the alterations of plasma proteins in CAD patients. These samples were fractionated by liquid chromatography and analyzed by tandem mass spectrometry (MS/MS). Based on the GO and Pathway enrichment analysis of differential proteins, we select 4 proteins for validation by ELISA method in the new patient cohort.

Among the total 3020 proteins identified from plasma by Human Proteome Organization, 345 proteins were labeled with cardiovascular-related functions [10] including CAD. Heatmap from Figure 1 showed that PF4 and sFRP1 had relative similar expression patterns.

Platelet factor 4 (PF4), a 70-amino acid protein, is stored in the alpha-granules of platelet and is released during platelet activation. PF4 plays a role in in thrombotic disease or inflammation progress and has anti-heparin activity [11], may promote the accumulation of deleterious lipoproteins [12] and may promote atherosclerosis [13].

In our study, the plasma level of PF4 was significantly up-regulated (*p* < 0.05). Given the covariate influence of age, gender, BMI, hypertension, DM, hyperlipidemia, hemoglobin and uric acid, there was a significant positive correlation between PF4 and CAD (Table 3). In consideration of the in vivo platelet activation that could be induced by heparin, PF4 was not considered as a biomarker for cardiovascular disorder or thrombus formation [14–17]. However, in the present study, ROC test showed that PF4 could act as a biomarker for the diagnosis of severe CAD (triple-vessel disease with/without left main stenosis) requiring CABG procedure. Furthermore, gender does not affect the levels of PF4 in CABG patients (Figure 5, Table 5), suggesting that the elevation of PF4 in CABG patients is true in both genders.

Frizzled proteins (fz) are 7-pass transmembrane proteins, the extracellular part of which might be the ligand-binding domain of Wnt proteins. Members of the Wnt/fz signal-transduction pathway are involved in cardiac hypertrophy and myocardial repair after myocardial infarction [18]. Secreted frizzled-related proteins (sFRPs) have a cysteine-rich domain similar to fz but lacking transmembrane domain. The sFRPs may therefore compete for Wnt binding and have a similar effect on cardiac pathology because their cysteine-rich domain. It has been demonstrated that sFRP1 is related to maturation in cardiomyocytes [19], proangiogenic effects [20], endothelial cells movement [21], and the post-infarction scar size [22].

In the present study, we detected an increased level of sFRP1 from the plasma of CAD patients (*p* < 0.05). In consideration of the effect of covariates, there was a moderate positive correlation between sFRP1 and CAD (Table 3). Partial correlation coefficient analysis showed that there was a positive relationship between myocardial infarction history and the level of sFRP1 (Table 4). These results suggest that sFRP1 and Wnt/fz signal-transduction pathway might participate in the pathologic progress of CAD and post-infarction myocardial remodeling. According to the result of ROC test in the present study, the best cut point of sFRP1 comes with a sensitivity of 92.4% and a specificity of 58.3%. Further, the level of sFRP1 in CABG group is affected by different gender (Figure 5), which was observed for the first time with much higher level of sFRP1 in the of female patients (Table 5).

Another protein validated by ELISA in the present study was blood coagulation factor XIII (F13). The main function of F13 is to strengthen fibrin polymers and protect them from the fibrinolysis [23] and related to protective effect on CAD and myocardial infarction [24]. However, it was validated by ELISA in the present study and therefore, the role of this protein in CABG patients need to be further investigated.

Some limitations of the study should be considered. To detect the proteomic changes, many important but high abundant proteins such as antibodies, apolipoproteins, protease inhibitors and coagulation factors are removed before the proteomic study [6–8, 25]. On the other hand, some lower concentration- plasma proteins that may be related to the pathology of CAD may not be able to be detected by the present techniques.

In summary, we for the first time have found 76 proteins differentially expressed in plasma samples from patients requiring CABG compared to control. Proteins involved in different physiological processes such as coagulation, platelet activation, complement pathway and Wnt/fz signal-transduction pathway may play an important role in the progress of CAD. The thrombotic disease or inflammation progress-related protein PF4 and sFRP1, a member of the Wnt/fz signal-transduction pathway, related to cardiac hypertrophy and myocardial repair after MI, are particularly important in severe CAD owing to the significant up-regulation in triple-vessel disease with/without left main stenosis. Among the identified altered proteins, PF4 may be developed as a biomarker for the diagnosis of the severity of the CAD (triple-vessel disease with/without left main stenosis) requiring CABG procedure.

## MATERIALS AND METHODS

### Study population

From April 22, 2013 to September 24, 2015, 320 patients who had diagnostic coronary angiography were enrolled and divided into two groups as CABG (160 patients with CAD who underwent CABG) and Control (control group, *n* = 160). All patients in CABG group had triple-vessel disease by coronary angiography and therefore underwent CABG procedure. Among the 160 CABG patients, 41 had left main disease. The control group was ruled out of CAD by angiography. To avoid the interference of the proteins that correlated with other diseases, patients with neurological disease (eg. cerebral vascular accident), severe pulmonary disease, renal dysfunction, liver dysfunction, active inflammation, infection, coagulation disorders, history of atrial fibrillation or thyroid dysfunction were excluded. Body mass index (BMI) was calculated as weight (kg) divided by the square of height (m^2^).

Clinical information was collected on age, gender, height, weight, history of myocardial infarction (MI), hypertension, hyperlipidemia and diabetes mellitus (DM). MI, hyperlipidemia, and DM were coded in a dichotomized manner. Hypertension was graded as normal, stage1, 2 and 3.

### Experimental design and protocols

Routine tests for patients were performed in clinical laboratory. Blood samples were collected before the angiographic procedure or CABG, and immediately centrifuged at 3500 rpm for 15 minutes. The plasma was collected carefully and stored at −80 degree until the analysis was carried out.

### Plasma high-abundance protein depletion

Plasma samples were processes to deplete the high abundance proteins using the ProteoMinerTM Kits (Bio-Rad Laboratories, Hercules, CA, USA).

### Solution digestion and iTRAQ labeling

#### iTRAQ technique and ELISA validation

The plasma samples of 20 males and 20 females in each group were marked with CABGM (CABG group, male), CABGF (CABG group, female), CM (control group, male) or CF (control group, female), respectively and analyzed with iTRAQ technique. The iTRAQ method and the design for ELISA validation followed the previously published procedures [9].

The samples eluted by Lysis buffer were reduced with 10 mmol/L DTT at 56°C for 60 min and then alkylated. The protein mixtures were precipitated by precooled acetone at −20°C overnight, and then centrifuged at 4°C, 30,000g for 30 min. The pellet was dissolved in 0.5 mmol/L TEAB (Applied Biosystems, Milan, Italy) and sonicated in ice. After a centrifuging at 4°C, 30,000g, the supernatant was used for liquid digestion with Trypsin Gold (Promega, Madison, WI, USA). The peptides were dried and reconstituted in 0.5 mmol/L TEAB and mixed with 70 ul of isopropanol. Samples were labeled with iTRAQ reagent (Applied Biosystems, Milan, Italy).

### SCX and LC-ESI-MS/MS analysis of labeled peptide

The peptides were dried and dissolved in 4 ml of buffer A (25 mmol/L NaH_2_PO_4_ in 25% CAN, pH 2.7). The sample was fractionated using cation-exchange chromatography (SCX) on a LC-20AB HPLC Pump system (Shimadzu, Kyoto, Japan). The peptides were eluted at a flow rate of 1mL/min with a gradient of buffer A for 10 min, 5-60% buffer B (25 mmol/L NaH_2_PO_4_ and 1 mol/L KCl in 25% ACN, pH 2.7) for 27 min, 60-100% buffer B for 1 min. The fractions were desalted and dried. Then, buffer A (5% ACN, 0.1% FA) was added to each dried fraction tube, and 10 ul supernatant of the re-dissolved solution was loaded on a LC-20AD nanoHPLC (Shimadzu, Kyoto, Japan) and separated over a 35 min gradient from 2 to 35% in 0.1% FA combined with 95% ACN. Data acquisition were performed with a TripleTOF 5600 System (AB SCIEX, Concord, ON).

### iTRAQ data analysis and bioinformatic analysis

Proteins identification was performed by using Mascot search engine (Matrix Science, London, UK; version 2.3.02). The quantitative protein ratios were weighted and normalized by the median ratio in Mascot. The fold changes of >1.2 (or < 0.8) and *p* value less than 0.05 were considered as significant.

### Human enzyme-linked immunosorbent assay (ELISA)

To verify the results of the proteomics, ELISA test for each individual patient in a new cohort of the CABG patients or in control was performed following iTRAQ procedure.

The ELISA Kit (CUSABIO BIOTECH, Life Sciences Advanced Technologies Inc, USA) was used to test the chosen proteins in plasma samples from CABG group (*n* = 120, male/femal = 61/59) and control (*n* = 120, male/female = 60/60).

### Ethics

The study protocol was approved by the ethical committee of TEDA International Cardiovascular Hospital and all patients were given informed consent. The clinical investigations were conducted according to the principles of the declaration of Helsinki.

### Statistics

Data analysis was performed using SPSS Statistics v 22.0 (IBM Corp, USA). Differences among groups were analyzed using ANOVA for continuous variables. Statistics significance was considered as *p* value less than 0.05. For proteins identified as significant also were tested with receiver operation characteristic (ROC) test. The ROC curve was generated in SPSS and the best differential point was detected according to the Youden Index (YI = sensitivity+specificity-1) [26, 27].

## SUPPLEMENTARY MATERIALS TABLE



## Abbreviations

CAD: coronary artery disease
BMI: body mass index
CABG: coronary artery bypass grafting
DM: diabetes mellitus
ELISA: enzyme-linked immunosorbent assay
F13: coagulation factor XIII
F13B: coagulation factor XIII B chain
fz: frizzled proteins
HIT: heparin-induced thrombocytopenia
iTRAQ: Isobaric tags for relative and absolute quantitation
MI: myocardial infarction
PF4: platelet factor 4
ROC: receiver operation characteristic
sFRP: secreted frizzled-related protein
sFRP1: secreted frizzled-related protein 1
SNV: single nucleotide variant
STEMI: ST-Elevated myocardial infarction
VSMC: vascular smooth muscle cell
YI: Youden Index
