# Supplementary material for: Plasma protein profiling in patients undergoing coronary artery bypass grafting surgery and clinical significance
Source: Oncotarget. 2017 Mar 18;8(36):60528–38. doi: 10.18632/oncotarget.16366 (PMC5601159; doi:10.18632/oncotarget.16366)
Supplement: Supplementary file 1 [file oncotarget-08-60528-s001.docx]

Supplementary Materials

Supplementary Table S1. Summary of proteins up- and down-regulated in CAD patients.

| Protein name | GenInfo Id^a^ and Accession no. | Unique Peptide | Cov (%) | M.W.^b^ | Score | Change Fold | GO Biological_Process | GO Molecular_Function | GO Cellular_Component |
| --- | --- | --- | --- | --- | --- | --- | --- | --- | --- |
| Midkine | gi\|159162699\|pdb\|1MKN\|A | 6 | 41 | 24178 | 95 | 2.67 | GO:0030421//defecation;GO:0021542//dentate gyrus development;GO:0021987//cerebral cortex development;GO:0009611//response to wounding;GO:0021681//cerebellar granular layer development;GO:0030154//cell differentiation;GO:0050795//regulation of behavior;GO:0051781//positive regulation of cell division;GO:0007165//signal transduction;GO:0001662//behavioral fear response;GO:0045893//positive regulation of transcription, DNA-dependent;GO:0030325//adrenal gland development;GO:0007614//short-term memory | GO:0008201//heparin binding;GO:0008083//growth factor activity | GO:0005576//extracellular region |
| Olfactomedin-like protein 3 | gi\|297664005\|ref\|XP_002810446.1\| | 7 | 16.2 | 44167 | 171 | 2.28 | GO:0007275//multicellular organismal development | - | GO:0005576//extracellular region |
| Myosin-reactive immunoglobulin light chain variable region (Fragment) | gi\|5921625\|gb\|AAD56272.1\| | 1 | 59.6 | 13027 | 1014 | 1.29 | - | - |  |
| Myosin-reactive immunoglobulin light chain variable region (Fragment) | gi\|5921615\|gb\|AAD56267.1\| | 1 | 36.1 | 13215 | 587 | 1.17 | - | - |  |
| cDNA FLJ34967 fis, clone NTONG2004690, highly similar to LIPOPROTEIN LIPASE (EC 3.1.1.34) | gi\|193787313\|dbj\|BAG52519.1\| | 6 | 15.6 | 63225 | 248 | 1.70 | GO:0034371//chylomicron remodeling;GO:0006644//phospholipid metabolic process;GO:0010890//positive regulation of sequestering of triglyceride;GO:0006633//fatty acid biosynthetic process;GO:0010886//positive regulation of cholesterol storage;GO:0034372//very-low-density lipoprotein particle remodeling;GO:0070328//triglyceride homeostasis;GO:0042157//lipoprotein metabolic process;GO:0019433//triglyceride catabolic process;GO:0010744//positive regulation of macrophage derived foam cell differentiation;GO:0019432//triglyceride biosynthetic process;GO:0042493//response to drug | GO:0004465//lipoprotein lipase activity;GO:0004620//phospholipase activity;GO:0017129//triglyceride binding;GO:0005102//receptor binding;GO:0004806//triglyceride lipase activity;GO:0008201//heparin binding | GO:0042627//chylomicron;GO:0009986//cell surface;GO:0031012//extracellular matrix;GO:0034361//very-low-density lipoprotein particle;GO:0031225//anchored to membrane;GO:0005886//plasma membrane |
| C4b-binding protein beta chain | gi\|4502505\|ref\|NP_000707.1\| | 7 | 30.6 | 35088 | 368 | 1.29 | GO:0006958//complement activation, classical pathway;GO:0045087//innate immune response;GO:0030449//regulation of complement activation;GO:0007596//blood coagulation | - | GO:0005576//extracellular region;GO:0005886//plasma membrane |
| Selenoprotein P (Fragment) | gi\|119576430\|gb\|EAW56026.1\| | 4 | 12.6 | 42698 | 444 | 1.12 | GO:0010269//response to selenium ion;GO:0001887//selenium compound metabolic process;GO:0006979//response to oxidative stress;GO:0009791//post-embryonic development;GO:0007420//brain development;GO:0040007//growth;GO:0019953//sexual reproduction;GO:0007626//locomotory behavior | GO:0008430//selenium binding | GO:0005615//extracellular space |
| Coagulation factor XIII B chain | gi\|110611237\|ref\|NP_001985.2\| | 14 | 29.2 | 91735 | 898 | 1.33 | GO:0007596//blood coagulation | - | GO:0005576//extracellular region |
| Platelet factor 4 | gi\|4505733\|ref\|NP_002610.1\| | 1 | 34.7 | 13861 | 479 | 1.56 | GO:0016525//negative regulation of angiogenesis;GO:0010628//positive regulation of gene expression;GO:0030168//platelet activation;GO:0032760//positive regulation of tumor necrosis factor production;GO:0043066//negative regulation of apoptotic process;GO:0010744//positive regulation of macrophage derived foam cell differentiation;GO:0006955//immune response;GO:0045918//negative regulation of cytolysis;GO:0019221//cytokine-mediated signaling pathway;GO:0045653//negative regulation of megakaryocyte differentiation;GO:0002576//platelet degranulation;GO:0045651//positive regulation of macrophage differentiation;GO:0045347//negative regulation of MHC class II biosynthetic process;GO:0030595//leukocyte chemotaxis | GO:0008201//heparin binding;GO:0008009//chemokine activity | GO:0031093//platelet alpha granule lumen;GO:0005615//extracellular space |
| Extracellular matrix protein 1 | gi\|221316614\|ref\|NP_004416.2\| | 6 | 11.3 | 67099 | 110 | 1.42 | GO:0001960//negative regulation of cytokine-mediated signaling pathway;GO:0001938//positive regulation of endothelial cell proliferation;GO:0006357//regulation of transcription from RNA polymerase II promoter;GO:0030502//negative regulation of bone mineralization;GO:0045766//positive regulation of angiogenesis;GO:2000404//regulation of T cell migration;GO:0043123//positive regulation of I-kappaB kinase/NF-kappaB cascade;GO:0006954//inflammatory response;GO:0002828//regulation of type 2 immune response;GO:0010466//negative regulation of peptidase activity | GO:0004871//signal transducer activity;GO:0043236//laminin binding;GO:0008022//protein C-terminus binding;GO:0002020//protease binding;GO:0005134//interleukin-2 receptor binding | GO:0005615//extracellular space;GO:0005578//proteinaceous extracellular matrix |
| Ig kappa chain V-III region B6 | gi\|125795\|sp\|P01619.1\|KV301_HUMAN | 4 | 31.5 | 12959 | 829 | 1.29 | - | - |  |
| Lactoferrin | gi\|193527456\|gb\|ACF19793.1\| | 18 | 30.7 | 94182 | 477 | 1.14 | GO:0042742//defense response to bacterium;GO:0090382//phagosome maturation;GO:0006508//proteolysis;GO:0033214//iron assimilation by chelation and transport;GO:0006959//humoral immune response;GO:0006826//iron ion transport;GO:0052572//response to host immune response | GO:0008199//ferric iron binding;GO:0005515//protein binding;GO:0004252//serine-type endopeptidase activity;GO:0008201//heparin binding | GO:0030141//secretory granule;GO:0097013//phagocytic vesicle lumen;GO:0005576//extracellular region |
| Secreted frizzled-related protein 1 | gi\|56117838\|ref\|NP_003003.3\| | 6 | 21.3 | 47834 | 141 | 3.13 | GO:0071504//cellular response to heparin;GO:0009267//cellular response to starvation;GO:0014070//response to organic cyclic compound;GO:2000054//negative regulation of Wnt receptor signaling pathway involved in dorsal/ventral axis specification;GO:0003401//axis elongation;GO:0060766//negative regulation of androgen receptor signaling pathway;GO:0002244//hematopoietic progenitor cell differentiation;GO:0060442//branching involved in prostate gland morphogenesis;GO:0032855//positive regulation of Rac GTPase activity;GO:0006309//apoptotic DNA fragmentation;GO:0045765//regulation of angiogenesis;GO:0046851//negative regulation of bone remodeling;GO:0051894//positive regulation of focal adhesion assembly;GO:0051496//positive regulation of stress fiber assembly;GO:0071456//cellular response to hypoxia;GO:0030336//negative regulation of cell migration;GO:0033689//negative regulation of osteoblast proliferation;GO:0008406//gonad development;GO:0071380//cellular response to prostaglandin E stimulus;GO:0071356//cellular response to tumor necrosis factor;GO:2000052//positive regulation of non-canonical Wnt receptor signaling pathway;GO:2000080//negative regulation of canonical Wnt receptor signaling pathway involved in controlling type B pancreatic cell proliferation;GO:0010719//negative regulation of epithelial to mesenchymal transition;GO:0071305//cellular response to vitamin D;GO:0045578//negative regulation of B cell differentiation;GO:0048147//negative regulation of fibroblast proliferation;GO:0060218//hematopoietic stem cell differentiation;GO:0001756//somitogenesis;GO:0001843//neural tube closure;GO:0030308//negative regulation of cell growth;GO:0045600//positive regulation of fat cell differentiation;GO:0071773//cellular response to BMP stimulus;GO:0050732//negative regulation of peptidyl-tyrosine phosphorylation;GO:0060071//Wnt receptor signaling pathway, planar cell polarity pathway;GO:0001649//osteoblast differentiation;GO:2000117//negative regulation of cysteine-type endopeptidase activity;GO:0007420//brain development;GO:0046676//negative regulation of insulin secretion;GO:0010564//regulation of cell cycle process;GO:0030307//positive regulation of cell growth;GO:0050680//negative regulation of epithelial cell proliferation;GO:0090263//positive regulation of canonical Wnt receptor signaling pathway;GO:0071392//cellular response to estradiol stimulus;GO:2000271//positive regulation of fibroblast apoptotic process;GO:0045893//positive regulation of transcription, DNA-dependent;GO:0044344//cellular response to fibroblast growth factor stimulus;GO:0030279//negative regulation of ossification;GO:0022601//menstrual cycle phase;GO:0071560//cellular response to transforming growth factor beta stimulus;GO:2000270//negative regulation of fibroblast apoptotic process;GO:0045880//positive regulation of smoothened signaling pathway;GO:0045892//negative regulation of transcription, DNA-dependent;GO:0042493//response to drug;GO:0050679//positive regulation of epithelial cell proliferation;GO:0071347//cellular response to interleukin-1 | GO:0030165//PDZ domain binding;GO:0004197//cysteine-type endopeptidase activity;GO:0042813//Wnt-activated receptor activity;GO:0042802//identical protein binding;GO:0005109//frizzled binding;GO:0008201//heparin binding;GO:0017147//Wnt-protein binding;GO:0008144//drug binding | GO:0042995//cell projection;GO:0009986//cell surface;GO:0005886//plasma membrane;GO:0005578//proteinaceous extracellular matrix;GO:0005829//cytosol;GO:0005615//extracellular space |
| SPARC-related modular calcium-binding protein 1 | gi\|441595540\|ref\|XP_004087249.1\| | 9 | 20.5 | 59786 | 293 | 2.06 | GO:0060173//limb development;GO:0045667//regulation of osteoblast differentiation;GO:0010811//positive regulation of cell-substrate adhesion;GO:0007165//signal transduction;GO:0001654//eye development;GO:0030198//extracellular matrix organization | GO:0050840//extracellular matrix binding;GO:0005509//calcium ion binding | GO:0005604//basement membrane |
| Hepatocyte growth factor activator | gi\|163931039\|pdb\|2R0L\|A | 5 | 10.4 | 78528 | 375 | 1.28 | GO:0006508//proteolysis | GO:0004252//serine-type endopeptidase activity | GO:0005615//extracellular space;GO:0005791//rough endoplasmic reticulum |
| ATP-binding cassette sub-family B member 9 | gi\|39992420\|gb\|AAH64384.1\| | 1 | 2.6 | 33165 | 104 | 1.19 | GO:0001916//positive regulation of T cell mediated cytotoxicity;GO:0002474//antigen processing and presentation of peptide antigen via MHC class I;GO:0015031//protein transport;GO:0015833//peptide transport;GO:0055085//transmembrane transport | GO:0046979//TAP2 binding;GO:0042288//MHC class I protein binding;GO:0046978//TAP1 binding;GO:0015421//oligopeptide-transporting ATPase activity;GO:0042605//peptide antigen binding;GO:0042803//protein homodimerization activity;GO:0005524//ATP binding;GO:0046980//tapasin binding | GO:0042825//TAP complex;GO:0005743//mitochondrial inner membrane;GO:0005765//lysosomal membrane;GO:0005769//early endosome;GO:0005886//plasma membrane |
| Ig kappa chain V-I region DEE | gi\|125761\|sp\|P01597.1\|KV105_HUMAN | 3 | 16.7 | 13897 | 800 | 1.54 | GO:0045087//innate immune response;GO:0006958//complement activation, classical pathway | GO:0003823//antigen binding | GO:0005886//plasma membrane;GO:0005576//extracellular region |
| Rheumatoid factor C6 light chain (Fragment) | gi\|185812\|gb\|AAA58972.1\| | 2 | 25 | 14764 | 560 | 1.20 | - | - |  |
| Immunoglobulin J chain | gi\|532598\|gb\|AAA58902.1\| | 5 | 35.2 | 21585 | 256 | 1.19 | GO:0006955//immune response | GO:0003823//antigen binding | GO:0005576//extracellular region |
| Myeloperoxidase | gi\|88180\|pir\|\|C28894 | 14 | 20.7 | 91781 | 668 | 1.70 | GO:0006952//defense response;GO:0042744//hydrogen peroxide catabolic process;GO:0055114//oxidation-reduction process;GO:0034374//low-density lipoprotein particle remodeling | GO:0046872//metal ion binding;GO:0004601//peroxidase activity;GO:0020037//heme binding;GO:0008201//heparin binding;GO:0003682//chromatin binding | GO:0005764//lysosome;GO:0030141//secretory granule;GO:0005615//extracellular space;GO:0005634//nucleus |
| Adipocyte enhancer-binding protein 1 | gi\|53692189\|ref\|NP_001120.3\| | 4 | 4.1 | 152883 | 109 | 1.78 | GO:0007517//muscle organ development;GO:0001501//skeletal system development | GO:0005488//binding;GO:0004180//carboxypeptidase activity;GO:0003700//sequence-specific DNA binding transcription factor activity | GO:0005615//extracellular space;GO:0031012//extracellular matrix |
| Complement C4B1a (Fragment) | gi\|68533033\|dbj\|BAE06071.1\| | 1 | 23.9 | 5960 | 138 | 2.20 | GO:0006958//complement activation, classical pathway;GO:0045087//innate immune response;GO:0030449//regulation of complement activation | GO:0004866//endopeptidase inhibitor activity | GO:0005615//extracellular space;GO:0005886//plasma membrane |
| Hepatocyte growth factor-like protein alpha chain | gi\|119585405\|gb\|EAW65001.1\| | 8 | 13.5 | 91508 | 137 | 1.36 | GO:0001569//patterning of blood vessels;GO:0007243//intracellular protein kinase cascade;GO:0001841//neural tube formation;GO:0007417//central nervous system development;GO:0046621//negative regulation of organ growth;GO:0090090//negative regulation of canonical Wnt receptor signaling pathway;GO:0000902//cell morphogenesis;GO:0046777//protein autophosphorylation;GO:0060215//primitive hemopoiesis;GO:0008285//negative regulation of cell proliferation;GO:0018105//peptidyl-serine phosphorylation;GO:0006917//induction of apoptosis;GO:0035329//hippo signaling cascade;GO:0060706//cell differentiation involved in embryonic placenta development;GO:0071902//positive regulation of protein serine/threonine kinase activity;GO:0006508//proteolysis;GO:0030216//keratinocyte differentiation;GO:0003157//endocardium development | GO:0004252//serine-type endopeptidase activity;GO:0004674//protein serine/threonine kinase activity;GO:0000287//magnesium ion binding;GO:0042803//protein homodimerization activity;GO:0008134//transcription factor binding;GO:0005524//ATP binding;GO:0043539//protein serine/threonine kinase activator activity | GO:0005576//extracellular region;GO:0005829//cytosol;GO:0005634//nucleus |
| Ig kappa chain C region (Fragment) | gi\|1827928\|pdb\|1CLY\|L | 2 | 80.4 | 14622 | 2413 | 1.61 | GO:0045087//innate immune response;GO:0006958//complement activation, classical pathway | GO:0003823//antigen binding | GO:0005886//plasma membrane;GO:0005576//extracellular region |
| cDNA FLJ56954, highly similar to Inter-alpha-trypsin inhibitor heavy chain H1 | gi\|221042196\|dbj\|BAH12775.1\| | 9 | 15.8 | 84584 | 582 | 1.30 | GO:0030212//hyaluronan metabolic process;GO:0045321//leukocyte activation | GO:0004867//serine-type endopeptidase inhibitor activity;GO:0005509//calcium ion binding | GO:0005576//extracellular region |
| Transforming growth factor, beta-induced, 68kDa variant (Fragment) | gi\|3282161\|gb\|AAC24944.1\| | 4 | 7.2 | 85574 | 155 | 1.26 | GO:0001525//angiogenesis;GO:0050896//response to stimulus;GO:0008283//cell proliferation;GO:0007601//visual perception;GO:0007162//negative regulation of cell adhesion | GO:0005178//integrin binding | GO:0005615//extracellular space;GO:0005886//plasma membrane;GO:0005578//proteinaceous extracellular matrix |
| Full-length cDNA clone CS0DD006YL02 of Neuroblastoma of Homo sapiens (human) | gi\|1335134\|emb\|CAA33069.1\| | 1 | 37.9 | 47597 | 8366 | 1.33 | GO:0006955//immune response | GO:0003823//antigen binding | GO:0005576//extracellular region;GO:0016021//integral to membrane;GO:0005886//plasma membrane |
| cDNA, FLJ93149, highly similar to Homo sapiens frizzled-related protein (FRZB), mRNA | gi\|189053445\|dbj\|BAG35611.1\| | 3 | 8.6 | 44184 | 108 | 4.33 | GO:0070367//negative regulation of hepatocyte differentiation;GO:0061053//somite development;GO:0090090//negative regulation of canonical Wnt receptor signaling pathway;GO:0007409//axonogenesis;GO:0001944//vasculature development;GO:0060029//convergent extension involved in organogenesis;GO:0008406//gonad development;GO:0008285//negative regulation of cell proliferation;GO:0061037//negative regulation of cartilage development;GO:0007420//brain development;GO:0010721//negative regulation of cell development;GO:0090103//cochlea morphogenesis;GO:0014033//neural crest cell differentiation;GO:0043065//positive regulation of apoptotic process;GO:0030308//negative regulation of cell growth;GO:0060056//mammary gland involution;GO:0045600//positive regulation of fat cell differentiation | GO:0030165//PDZ domain binding;GO:0042813//Wnt-activated receptor activity;GO:0017147//Wnt-protein binding | GO:0005615//extracellular space;GO:0032589//neuron projection membrane;GO:0005737//cytoplasm |
| cDNA, FLJ93141, highly similar to Homo sapiens coagulation factor XIII, A1 polypeptide (F13A1), mRNA | gi\|189053440\|dbj\|BAG35606.1\| | 19 | 28.3 | 95566 | 1294 | 1.47 | GO:0018149//peptide cross-linking;GO:0030168//platelet activation;GO:0002576//platelet degranulation | GO:0046872//metal ion binding;GO:0003810//protein-glutamine gamma-glutamyltransferase activity | GO:0031093//platelet alpha granule lumen;GO:0005576//extracellular region |
| Vh1-D-J3-region (Fragment) | gi\|292193\|gb\|AAA51013.1\| | 1 | 9.4 | 15986 | 118 | 1.22 | - | - |  |
| Plasma kallikrein heavy chain (Fragment) | gi\|78191798\|ref\|NP_000883.2\| | 13 | 21.4 | 91778 | 400 | 1.25 | GO:0007597//blood coagulation, intrinsic pathway;GO:0031639//plasminogen activation;GO:0022617//extracellular matrix disassembly;GO:0051919//positive regulation of fibrinolysis;GO:0006508//proteolysis;GO:0002542//Factor XII activation | GO:0016705//oxidoreductase activity, acting on paired donors, with incorporation or reduction of molecular oxygen;GO:0009055//electron carrier activity;GO:0004497//monooxygenase activity;GO:0005515//protein binding;GO:0020037//heme binding;GO:0004252//serine-type endopeptidase activity;GO:0005506//iron ion binding | GO:0005615//extracellular space;GO:0005886//plasma membrane |
| Spondin-1 | gi\|110347423\|ref\|NP_006099.2\| | 3 | 3.8 | 109355 | 81 | 2.10 | GO:0007155//cell adhesion | GO:0005515//protein binding | GO:0005615//extracellular space;GO:0005578//proteinaceous extracellular matrix |
| Coagulation factor XII-Mie | gi\|24899162\|dbj\|BAC23095.1\| | 7 | 14.5 | 76113 | 365 | 1.52 | GO:0007597//blood coagulation, intrinsic pathway;GO:0030194//positive regulation of blood coagulation;GO:0051788//response to misfolded protein;GO:0045087//innate immune response;GO:0051919//positive regulation of fibrinolysis;GO:0016540//protein autoprocessing;GO:0006508//proteolysis;GO:0002542//Factor XII activation;GO:0010756//positive regulation of plasminogen activation | GO:0051787//misfolded protein binding;GO:0004252//serine-type endopeptidase activity | GO:0005615//extracellular space;GO:0005886//plasma membrane |
| Myosin-reactive immunoglobulin heavy chain variable region (Fragment) | gi\|5921605\|gb\|AAD56262.1\| | 1 | 24.4 | 17289 | 129 | 1.30 | - | - |  |
| Intelectin 1 | gi\|20377087\|gb\|AAM20741.1\|AF271386_1 | 5 | 16.9 | 39480 | 147 | 1.15 | GO:0046326//positive regulation of glucose import;GO:0007165//signal transduction;GO:0001934//positive regulation of protein phosphorylation;GO:0009624//response to nematode | GO:0005102//receptor binding;GO:0030246//carbohydrate binding | GO:0031526//brush border membrane;GO:0045121//membrane raft;GO:0005576//extracellular region;GO:0031225//anchored to membrane |
| Procollagen C-endopeptidase enhancer | gi\|51094573\|gb\|EAL23825.1\| | 8 | 25.8 | 55476 | 590 | 0.80 | GO:0010952//positive regulation of peptidase activity;GO:0007275//multicellular organismal development;GO:0006508//proteolysis | GO:0008201//heparin binding;GO:0005518//collagen binding;GO:0016504//peptidase activator activity | GO:0005615//extracellular space;GO:0031012//extracellular matrix |
| TIMP metallopeptidase inhibitor 2, isoform CRA_b | gi\|1517893\|gb\|AAC50729.1\| | 2 | 8.4 | 26475 | 88 | 0.72 | GO:0030814//regulation of cAMP metabolic process;GO:0022617//extracellular matrix disassembly;GO:0045664//regulation of neuron differentiation;GO:0071310//cellular response to organic substance;GO:0008285//negative regulation of cell proliferation;GO:0043086//negative regulation of catalytic activity;GO:0043408//regulation of MAPK cascade;GO:0043085//positive regulation of catalytic activity | GO:0008047//enzyme activator activity;GO:0046872//metal ion binding;GO:0005178//integrin binding;GO:0008191//metalloendopeptidase inhibitor activity | GO:0005604//basement membrane;GO:0009986//cell surface |
| Carbamoylphosphate synthetase I | gi\|56378239\|dbj\|BAD74209.1\| | 7 | 5.4 | 197856 | 125 | 0.35 | GO:0032496//response to lipopolysaccharide;GO:0019240//citrulline biosynthetic process;GO:0032094//response to food;GO:0005980//glycogen catabolic process;GO:0000050//urea cycle;GO:0071400//cellular response to oleic acid;GO:0071548//response to dexamethasone stimulus;GO:0007494//midgut development;GO:0060416//response to growth hormone stimulus;GO:0019433//triglyceride catabolic process;GO:0071377//cellular response to glucagon stimulus;GO:0070365//hepatocyte differentiation;GO:0010043//response to zinc ion;GO:0042594//response to starvation;GO:0042493//response to drug;GO:0043200//response to amino acid stimulus;GO:0050667//homocysteine metabolic process;GO:0006543//glutamine catabolic process;GO:0071320//cellular response to cAMP;GO:0046209//nitric oxide metabolic process;GO:0045909//positive regulation of vasodilation;GO:0055081//anion homeostasis;GO:0009636//response to toxic substance;GO:0044344//cellular response to fibroblast growth factor stimulus;GO:0070409//carbamoyl phosphate biosynthetic process | GO:0004175//endopeptidase activity;GO:0005509//calcium ion binding;GO:0005543//phospholipid binding;GO:0016595//glutamate binding;GO:0072341//modified amino acid binding;GO:0032403//protein complex binding;GO:0005524//ATP binding;GO:0004087//carbamoyl-phosphate synthase (ammonia) activity | GO:0005743//mitochondrial inner membrane;GO:0042645//mitochondrial nucleoid;GO:0005730//nucleolus;GO:0043234//protein complex |
| Alpha-1-acid glycoprotein 2 | gi\|29170378\|emb\|CAA29873.2\| | 2 | 14.4 | 28132 | 139 | 0.73 | GO:0002682//regulation of immune system process;GO:0006953//acute-phase response;GO:0006810//transport | GO:0005515//protein binding | GO:0005615//extracellular space |
| Vitamin D-binding protein | gi\|324021745\|ref\|NP_001191236.1\| | 8 | 18.5 | 67663 | 146 | 0.49 | GO:0050728//negative regulation of inflammatory response;GO:0043687//post-translational protein modification;GO:0035307//positive regulation of protein dephosphorylation;GO:0009790//embryo development;GO:0007596//blood coagulation;GO:0071356//cellular response to tumor necrosis factor;GO:0046512//sphingosine biosynthetic process;GO:0017187//peptidyl-glutamic acid carboxylation;GO:0051180//vitamin transport;GO:0046513//ceramide biosynthetic process;GO:0043407//negative regulation of MAP kinase activity;GO:0042359//vitamin D metabolic process;GO:0008219//cell death;GO:0005975//carbohydrate metabolic process;GO:0032571//response to vitamin K;GO:0023021//termination of signal transduction;GO:0007565//female pregnancy;GO:0032355//response to estradiol stimulus;GO:0032715//negative regulation of interleukin-6 production;GO:0006680//glucosylceramide catabolic process;GO:0007595//lactation;GO:0006644//phospholipid metabolic process;GO:0007040//lysosome organization | GO:0005102//receptor binding;GO:0008488//gamma-glutamyl carboxylase activity;GO:0004348//glucosylceramidase activity;GO:0005499//vitamin D binding;GO:0051183//vitamin transporter activity;GO:0043169//cation binding;GO:0003779//actin binding | GO:0005829//cytosol;GO:0005615//extracellular space;GO:0030424//axon;GO:0043202//lysosomal lumen;GO:0048471//perinuclear region of cytoplasm;GO:0016021//integral to membrane;GO:0005765//lysosomal membrane;GO:0005789//endoplasmic reticulum membrane |
| Myosin-reactive immunoglobulin heavy chain variable region (Fragment) | gi\|5921603\|gb\|AAD56261.1\| | 2 | 23.3 | 14841 | 137 | 0.82 | - | - |  |
| Glyceraldehyde-3-phosphate dehydrogenase | gi\|54303910\|gb\|AAV33305.1\| | 6 | 16.7 | 44411 | 231 | 0.22 | GO:0051402//neuron apoptotic process;GO:0055114//oxidation-reduction process;GO:0035606//peptidyl-cysteine S-trans-nitrosylation;GO:0050821//protein stabilization;GO:0000226//microtubule cytoskeleton organization;GO:0006096//glycolysis;GO:0006094//gluconeogenesis;GO:0044281//small molecule metabolic process | GO:0051287//NAD binding;GO:0008017//microtubule binding;GO:0035605//peptidyl-cysteine S-nitrosylase activity;GO:0004365//glyceraldehyde-3-phosphate dehydrogenase (NAD+) (phosphorylating) activity;GO:0050661//NADP binding | GO:0005829//cytosol;GO:0015630//microtubule cytoskeleton;GO:0048471//perinuclear region of cytoplasm;GO:0005811//lipid particle;GO:0016020//membrane;GO:0005634//nucleus |
| Adipocyte plasma membrane-associated protein (Fragment) | gi\|119630519\|gb\|EAX10114.1\| | 5 | 13.4 | 50834 | 137 | 0.59 | GO:0009058//biosynthetic process | GO:0004064//arylesterase activity;GO:0016844//strictosidine synthase activity | GO:0016021//integral to membrane;GO:0009986//cell surface |
| Properdin | gi\|73535361\|pdb\|1W0R\|A | 5 | 13.6 | 59531 | 237 | 0.32 | GO:0042742//defense response to bacterium;GO:0006957//complement activation, alternative pathway;GO:0030449//regulation of complement activation | - | GO:0005615//extracellular space;GO:0031012//extracellular matrix |
| A disintegrin and metalloproteinase with thrombospondin motifs 13 | gi\|119608489\|gb\|EAW88083.1\| | 17 | 13.1 | 165786 | 431 | 0.84 | GO:0030168//platelet activation;GO:0007229//integrin-mediated signaling pathway;GO:0043171//peptide catabolic process;GO:0007160//cell-matrix adhesion;GO:0006508//proteolysis;GO:0034341//response to interferon-gamma;GO:0070670//response to interleukin-4;GO:0034612//response to tumor necrosis factor;GO:0016485//protein processing;GO:0009100//glycoprotein metabolic process | GO:0008270//zinc ion binding;GO:0005509//calcium ion binding;GO:0004222//metalloendopeptidase activity;GO:0005178//integrin binding | GO:0005615//extracellular space;GO:0009986//cell surface;GO:0005578//proteinaceous extracellular matrix |
| N-acetylmuramoyl-L-alanine amidase | gi\|156616294\|ref\|NP_443122.3\| | 8 | 20 | 66703 | 447 | 0.49 | GO:0032827//negative regulation of natural killer cell differentiation involved in immune response;GO:0044117//growth of symbiont in host;GO:0045087//innate immune response;GO:0001519//peptide amidation;GO:0009253//peptidoglycan catabolic process;GO:0032689//negative regulation of interferon-gamma production;GO:0016045//detection of bacterium;GO:0050830//defense response to Gram-positive bacterium | GO:0008270//zinc ion binding;GO:0008745//N-acetylmuramoyl-L-alanine amidase activity;GO:0042834//peptidoglycan binding;GO:0016019//peptidoglycan receptor activity | GO:0005622//intracellular;GO:0005576//extracellular region;GO:0016020//membrane |
| cDNA, FLJ93024, highly similar to Homo sapiens EGF-containing fibulin-like extracellular matrix protein 1 (EFEMP1), transcript variant 1, mRNA | gi\|189069185\|dbj\|BAG35523.1\| | 7 | 16 | 60522 | 511 | 0.75 | GO:0018108//peptidyl-tyrosine phosphorylation;GO:0006355//regulation of transcription, DNA-dependent;GO:0007601//visual perception;GO:0032331//negative regulation of chondrocyte differentiation;GO:0007173//epidermal growth factor receptor signaling pathway | GO:0005509//calcium ion binding;GO:0005006//epidermal growth factor-activated receptor activity;GO:0008083//growth factor activity;GO:0005154//epidermal growth factor receptor binding | GO:0005615//extracellular space;GO:0005578//proteinaceous extracellular matrix |
| Isocitrate dehydrogenase 1 (Fragment) | gi\|28178825\|ref\|NP_005887.2\| | 2 | 5.9 | 51521 | 42 | 0.29 | GO:0006979//response to oxidative stress;GO:0006097//glyoxylate cycle;GO:0006103//2-oxoglutarate metabolic process;GO:0006749//glutathione metabolic process;GO:0006099//tricarboxylic acid cycle;GO:0006740//NADPH regeneration;GO:0006102//isocitrate metabolic process;GO:0044255//cellular lipid metabolic process | GO:0051287//NAD binding;GO:0005102//receptor binding;GO:0004450//isocitrate dehydrogenase (NADP+) activity;GO:0000287//magnesium ion binding;GO:0042803//protein homodimerization activity | GO:0005829//cytosol;GO:0005739//mitochondrion;GO:0005782//peroxisomal matrix |
| Complement C3 | gi\|115298678\|ref\|NP_000055.2\| | 81 | 52.9 | 223249 | 19036 | 0.46 | GO:0001934//positive regulation of protein phosphorylation;GO:0006957//complement activation, alternative pathway;GO:0010884//positive regulation of lipid storage;GO:0070374//positive regulation of ERK1 and ERK2 cascade;GO:0032026//response to magnesium ion;GO:0050766//positive regulation of phagocytosis;GO:0045766//positive regulation of angiogenesis;GO:0045745//positive regulation of G-protein coupled receptor protein signaling pathway;GO:0032570//response to progesterone stimulus;GO:0051384//response to glucocorticoid stimulus;GO:0001798//positive regulation of type IIa hypersensitivity;GO:0002507//tolerance induction;GO:0007596//blood coagulation;GO:0048639//positive regulation of developmental growth;GO:0010575//positive regulation vascular endothelial growth factor production;GO:0006935//chemotaxis;GO:0006958//complement activation, classical pathway;GO:0010828//positive regulation of glucose transport;GO:0010866//regulation of triglyceride biosynthetic process;GO:0001970//positive regulation of activation of membrane attack complex;GO:0032355//response to estradiol stimulus | GO:0048037//cofactor binding;GO:0031715//C5L2 anaphylatoxin chemotactic receptor binding;GO:0008289//lipid binding;GO:0004866//endopeptidase inhibitor activity | GO:0005615//extracellular space;GO:0005886//plasma membrane |
| Protein AMBP | gi\|307077\|gb\|AAA59194.1\| | 9 | 36.4 | 45666 | 1160 | 0.80 | GO:0042167//heme catabolic process;GO:0046329//negative regulation of JNK cascade;GO:0050777//negative regulation of immune response;GO:0018298//protein-chromophore linkage;GO:0007565//female pregnancy;GO:0007155//cell adhesion;GO:0019048//virus-host interaction | GO:0004867//serine-type endopeptidase inhibitor activity;GO:0019855//calcium channel inhibitor activity;GO:0046904//calcium oxalate binding;GO:0020037//heme binding;GO:0019862//IgA binding;GO:0042803//protein homodimerization activity;GO:0036094//small molecule binding | GO:0005576//extracellular region;GO:0005886//plasma membrane |
| Protein S100-A8 | gi\|29888\|emb\|CAA68390.1\| | 4 | 29 | 14839 | 242 | 0.17 | GO:0006935//chemotaxis;GO:0002526//acute inflammatory response;GO:0042060//wound healing;GO:0002544//chronic inflammatory response;GO:0010043//response to zinc ion;GO:0045471//response to ethanol;GO:0032496//response to lipopolysaccharide | GO:0005515//protein binding;GO:0005509//calcium ion binding | GO:0005737//cytoplasm;GO:0005856//cytoskeleton;GO:0005615//extracellular space;GO:0005886//plasma membrane |
| von Willebrand factor | gi\|442564354\|emb\|CCQ25771.1\| | 1 | 16.8 | 341812 | 1494 | 0.54 | GO:0007155//cell adhesion;GO:0007596//blood coagulation | - | GO:0005578//proteinaceous extracellular matrix |
| Mannose-binding protein C | gi\|4557739\|ref\|NP_000233.1\| | 4 | 16.9 | 32610 | 131 | 0.53 | GO:0006979//response to oxidative stress;GO:0008228//opsonization;GO:0050766//positive regulation of phagocytosis;GO:0006958//complement activation, classical pathway;GO:0051873//killing by host of symbiont cells;GO:0001867//complement activation, lectin pathway;GO:0006953//acute-phase response;GO:0050830//defense response to Gram-positive bacterium;GO:0044130//negative regulation of growth of symbiont in host | GO:0051635//bacterial cell surface binding;GO:0005537//mannose binding;GO:0043499//eukaryotic cell surface binding;GO:0005102//receptor binding;GO:0048306//calcium-dependent protein binding | GO:0005581//collagen;GO:0005615//extracellular space |
| Alpha-enolase | gi\|301030821\|gb\|ADK47995.1\| | 9 | 29.7 | 59345 | 313 | 0.29 | GO:0009615//response to virus;GO:0030308//negative regulation of cell growth;GO:0000122//negative regulation of transcription from RNA polymerase II promoter;GO:0006096//glycolysis;GO:0006094//gluconeogenesis;GO:0044281//small molecule metabolic process | GO:0003677//DNA binding;GO:0003714//transcription corepressor activity;GO:0005515//protein binding;GO:0000287//magnesium ion binding;GO:0003700//sequence-specific DNA binding transcription factor activity;GO:0004634//phosphopyruvate hydratase activity | GO:0031430//M band;GO:0000015//phosphopyruvate hydratase complex;GO:0009986//cell surface;GO:0005634//nucleus;GO:0005886//plasma membrane |
| Complement C4-A | gi\|401871711\|pdb\|4FXG\|A | 2 | 41.9 | 217989 | 25479 | 0.78 | GO:0006958//complement activation, classical pathway;GO:0045087//innate immune response;GO:0030449//regulation of complement activation | GO:0004866//endopeptidase inhibitor activity | GO:0005615//extracellular space;GO:0005886//plasma membrane |
| Hemopexin | gi\|1335098\|emb\|CAA26382.1\| | 11 | 31.6 | 59077 | 500 | 0.81 | GO:0006879//cellular iron ion homeostasis;GO:0002639//positive regulation of immunoglobulin production;GO:0042168//heme metabolic process;GO:0019048//virus-host interaction;GO:0002925//positive regulation of humoral immune response mediated by circulating immunoglobulin;GO:0015886//heme transport;GO:0020027//hemoglobin metabolic process;GO:0060335//positive regulation of interferon-gamma-mediated signaling pathway;GO:0042511//positive regulation of tyrosine phosphorylation of Stat1 protein | GO:0005515//protein binding;GO:0046872//metal ion binding;GO:0015232//heme transporter activity | GO:0005615//extracellular space |
| cDNA FLJ53009, highly similar to Calreticulin | gi\|355755517\|gb\|EHH59264.1\| | 4 | 21.2 | 31440 | 143 | 0.78 | GO:0055007//cardiac muscle cell differentiation;GO:0045665//negative regulation of neuron differentiation;GO:0002479//antigen processing and presentation of exogenous peptide antigen via MHC class I, TAP-dependent;GO:0008284//positive regulation of cell proliferation;GO:0043687//post-translational protein modification;GO:0030866//cortical actin cytoskeleton organization;GO:0007283//spermatogenesis;GO:0017148//negative regulation of translation;GO:0033574//response to testosterone stimulus;GO:0045740//positive regulation of DNA replication;GO:0002502//peptide antigen assembly with MHC class I protein complex;GO:0042981//regulation of apoptotic process;GO:0006611//protein export from nucleus;GO:0042921//glucocorticoid receptor signaling pathway;GO:0051208//sequestering of calcium ion;GO:0000122//negative regulation of transcription from RNA polymerase II promoter;GO:0007050//cell cycle arrest;GO:0071285//cellular response to lithium ion;GO:0022417//protein maturation by protein folding;GO:0040020//regulation of meiosis;GO:0050821//protein stabilization;GO:0050766//positive regulation of phagocytosis;GO:0048387//negative regulation of retinoic acid receptor signaling pathway;GO:0006987//activation of signaling protein activity involved in unfolded protein response;GO:0045787//positive regulation of cell cycle;GO:0033144//negative regulation of intracellular steroid hormone receptor signaling pathway;GO:0010628//positive regulation of gene expression;GO:0018279//protein N-linked glycosylation via asparagine;GO:0032355//response to estradiol stimulus;GO:0042493//response to drug;GO:0090398//cellular senescence;GO:0034504//protein localization to nucleus | GO:0051087//chaperone binding;GO:0005506//iron ion binding;GO:0051082//unfolded protein binding;GO:0031625//ubiquitin protein ligase binding;GO:0042562//hormone binding;GO:0044183//protein binding involved in protein folding;GO:0030246//carbohydrate binding;GO:0001849//complement component C1q binding;GO:0005178//integrin binding;GO:0003729//mRNA binding;GO:0050681//androgen receptor binding;GO:0042277//peptide binding;GO:0008270//zinc ion binding;GO:0003677//DNA binding;GO:0005509//calcium ion binding | GO:0005829//cytosol;GO:0005615//extracellular space;GO:0005844//polysome;GO:0005578//proteinaceous extracellular matrix;GO:0001669//acrosomal vesicle;GO:0048471//perinuclear region of cytoplasm;GO:0042824//MHC class I peptide loading complex;GO:0005794//Golgi apparatus;GO:0009897//external side of plasma membrane;GO:0071556//integral to lumenal side of endoplasmic reticulum membrane;GO:0033018//sarcoplasmic reticulum lumen;GO:0005634//nucleus |
| Biotinidase | gi\|4557373\|ref\|NP_000051.1\| | 2 | 4.6 | 68090 | 109 | 0.34 | GO:0007417//central nervous system development;GO:0008544//epidermis development;GO:0006807//nitrogen compound metabolic process | GO:0004075//biotin carboxylase activity;GO:0047708//biotinidase activity | GO:0005730//nucleolus;GO:0005615//extracellular space;GO:0045177//apical part of cell;GO:0043204//perikaryon |
| cDNA, FLJ93695, highly similar to Homo sapiens serpin peptidase inhibitor, clade A (alpha-1 antiproteinase, antitrypsin), member 4 (SERPINA4), mRNA | gi\|189053760\|dbj\|BAG36012.1\| | 8 | 20.6 | 57184 | 304 | 0.87 | GO:0010951//negative regulation of endopeptidase activity;GO:0030162//regulation of proteolysis | GO:0004867//serine-type endopeptidase inhibitor activity | GO:0005615//extracellular space |
| Retinoic acid receptor responder protein 2 | gi\|4506427\|ref\|NP_002880.1\| | 4 | 32.5 | 22598 | 238 | 0.78 | GO:0050873//brown fat cell differentiation;GO:0048566//embryonic digestive tract development;GO:0001523//retinoid metabolic process;GO:0001701//in utero embryonic development;GO:0010759//positive regulation of macrophage chemotaxis | GO:0005102//receptor binding | GO:0031012//extracellular matrix;GO:0005576//extracellular region |
| Lumican variant (Fragment) | gi\|62897915\|dbj\|BAD96897.1\| | 4 | 13 | 46640 | 55 | 0.69 | GO:0070848//response to growth factor stimulus;GO:0051216//cartilage development;GO:0018146//keratan sulfate biosynthetic process;GO:0005975//carbohydrate metabolic process;GO:0007601//visual perception;GO:0014070//response to organic cyclic compound;GO:0030199//collagen fibril organization;GO:0044281//small molecule metabolic process;GO:0042340//keratan sulfate catabolic process;GO:0045944//positive regulation of transcription from RNA polymerase II promoter | GO:0005201//extracellular matrix structural constituent;GO:0005518//collagen binding | GO:0005615//extracellular space;GO:0005796//Golgi lumen;GO:0043202//lysosomal lumen;GO:0005583//fibrillar collagen |
| Proprotein convertase subtilisin/kexin type 9 | gi\|354459525\|pdb\|3P5B\|P | 7 | 13.3 | 80589 | 292 | 0.73 | GO:0043086//negative regulation of catalytic activity;GO:0032869//cellular response to insulin stimulus;GO:0001822//kidney development;GO:0032803//regulation of low-density lipoprotein particle receptor catabolic process;GO:0010989//negative regulation of low-density lipoprotein particle clearance;GO:0001920//negative regulation of receptor recycling;GO:0008203//cholesterol metabolic process;GO:0042157//lipoprotein metabolic process;GO:0016540//protein autoprocessing;GO:0007041//lysosomal transport;GO:0006917//induction of apoptosis;GO:0010469//regulation of receptor activity;GO:0006508//proteolysis;GO:0006641//triglyceride metabolic process;GO:0001889//liver development;GO:0043525//positive regulation of neuron apoptotic process;GO:0006644//phospholipid metabolic process;GO:0009267//cellular response to starvation;GO:0002092//positive regulation of receptor internalization;GO:0042632//cholesterol homeostasis;GO:0030182//neuron differentiation | GO:0034190//apolipoprotein receptor binding;GO:0019871//sodium channel inhibitor activity;GO:0004252//serine-type endopeptidase activity;GO:0034189//very-low-density lipoprotein particle binding;GO:0043621//protein self-association;GO:0050750//low-density lipoprotein particle receptor binding;GO:0070326//very-low-density lipoprotein particle receptor binding;GO:0030169//low-density lipoprotein particle binding;GO:0042802//identical protein binding;GO:0034185//apolipoprotein binding | GO:0005615//extracellular space;GO:0005764//lysosome;GO:0005794//Golgi apparatus;GO:0005770//late endosome;GO:0005769//early endosome;GO:0048471//perinuclear region of cytoplasm;GO:0005791//rough endoplasmic reticulum;GO:0031232//extrinsic to external side of plasma membrane |
| Alcohol dehydrogenase 4 (Class II), pi polypeptide, isoform CRA_a | gi\|119626493\|gb\|EAX06088.1\| | 5 | 13.7 | 51465 | 165 | 0.32 | GO:0042572//retinol metabolic process;GO:0042375//quinone cofactor metabolic process;GO:0006069//ethanol oxidation;GO:0006805//xenobiotic metabolic process;GO:0046164//alcohol catabolic process;GO:0006081//cellular aldehyde metabolic process | GO:0051287//NAD binding;GO:0004032//alditol:NADP+ 1-oxidoreductase activity;GO:0008270//zinc ion binding;GO:0019841//retinol binding;GO:0004745//retinol dehydrogenase activity;GO:0005503//all-trans retinal binding;GO:0003960//NADPH:quinone reductase activity;GO:0004024//alcohol dehydrogenase activity, zinc-dependent;GO:0019115//benzaldehyde dehydrogenase activity | GO:0005829//cytosol;GO:0015630//microtubule cytoskeleton |
| Apolipoprotein(a) | gi\|114062\|sp\|P08519.1\|APOA_HUMAN | 22 | 6.9 | 522647 | 1106 | 0.43 | GO:0006810//transport | GO:0005515//protein binding;GO:0008236//serine-type peptidase activity | - |
| cDNA, FLJ94213, highly similar to Homo sapiens pregnancy-zone protein (PZP), mRNA | gi\|189069365\|dbj\|BAG36397.1\| | 18 | 20.2 | 187153 | 939 | 0.73 | GO:0007565//female pregnancy;GO:0010951//negative regulation of endopeptidase activity | GO:0004867//serine-type endopeptidase inhibitor activity | GO:0005615//extracellular space |
| Protein S100 | gi\|189053201\|dbj\|BAG34823.1\| | 2 | 17.5 | 16909 | 69 | 0.13 | GO:0007267//cell-cell signaling;GO:0045471//response to ethanol;GO:0006935//chemotaxis;GO:0007165//signal transduction;GO:0010043//response to zinc ion;GO:0002544//chronic inflammatory response;GO:0032496//response to lipopolysaccharide | GO:0005509//calcium ion binding;GO:0004871//signal transducer activity;GO:0005515//protein binding | GO:0005856//cytoskeleton;GO:0005730//nucleolus;GO:0005615//extracellular space;GO:0005737//cytoplasm;GO:0005886//plasma membrane |
| Thyroxine-binding globulin | gi\|205277441\|ref\|NP_000345.2\| | 4 | 9.4 | 55459 | 94 | 0.46 | GO:0034695//response to prostaglandin E stimulus;GO:0010951//negative regulation of endopeptidase activity;GO:0042493//response to drug;GO:0043434//response to peptide hormone stimulus;GO:0033189//response to vitamin A;GO:0007568//aging;GO:0009791//post-embryonic development;GO:0030162//regulation of proteolysis;GO:0051412//response to corticosterone stimulus | GO:0004867//serine-type endopeptidase inhibitor activity;GO:0042562//hormone binding | GO:0005615//extracellular space |
| cDNA FLJ53063, highly similar to Tubulin beta-7 chain | gi\|410905733\|ref\|XP_003966346.1\| | 6 | 19.6 | 39890 | 140 | 0.54 | - | - |  |
| Secreted phosphoprotein 24 | gi\|5902118\|ref\|NP_008875.1\| | 5 | 26.5 | 27041 | 234 | 0.78 | GO:0046849//bone remodeling;GO:0010951//negative regulation of endopeptidase activity;GO:0001501//skeletal system development | GO:0004866//endopeptidase inhibitor activity | GO:0043234//protein complex;GO:0005576//extracellular region |
| cDNA FLJ52842, highly similar to Actin, cytoplasmic 1 | gi\|194385944\|dbj\|BAG65347.1\| | 9 | 30.2 | 45627 | 567 | 0.37 | GO:0007411//axon guidance;GO:0034332//adherens junction organization;GO:0006928//cellular component movement;GO:0051592//response to calcium ion;GO:0045214//sarcomere organization;GO:0034329//cell junction assembly;GO:0007596//blood coagulation;GO:0051086//chaperone mediated protein folding independent of cofactor | GO:0019901//protein kinase binding;GO:0050998//nitric-oxide synthase binding;GO:0019894//kinesin binding;GO:0030957//Tat protein binding;GO:0042802//identical protein binding;GO:0005524//ATP binding;GO:0005200//structural constituent of cytoskeleton | GO:0030863//cortical cytoskeleton;GO:0031941//filamentous actin;GO:0005829//cytosol;GO:0030016//myofibril;GO:0030424//axon;GO:0014069//postsynaptic density;GO:0070688//MLL5-L complex;GO:0035267//NuA4 histone acetyltransferase complex;GO:0030529//ribonucleoprotein complex |
| Pyruvate kinase | gi\|426379615\|ref\|XP_004056487.1\| | 6 | 11.9 | 70098 | 171 | 0.25 | GO:0016310//phosphorylation;GO:0044281//small molecule metabolic process;GO:0006096//glycolysis;GO:0012501//programmed cell death | GO:0004743//pyruvate kinase activity;GO:0005515//protein binding;GO:0030955//potassium ion binding;GO:0005524//ATP binding;GO:0000287//magnesium ion binding | GO:0005886//plasma membrane;GO:0005634//nucleus;GO:0005829//cytosol;GO:0005739//mitochondrion |
| Annexin | gi\|194390972\|dbj\|BAG60604.1\| | 4 | 12.9 | 53935 | 114 | 0.40 | GO:0007109//cytokinesis, completion of separation;GO:0007049//cell cycle;GO:0006909//phagocytosis;GO:0051592//response to calcium ion | GO:0008429//phosphatidylethanolamine binding;GO:0005509//calcium ion binding;GO:0005544//calcium-dependent phospholipid binding;GO:0044548//S100 protein binding;GO:0048306//calcium-dependent protein binding | GO:0042582//azurophil granule;GO:0030496//midbody;GO:0042470//melanosome;GO:0042581//specific granule;GO:0005819//spindle;GO:0045335//phagocytic vesicle;GO:0005635//nuclear envelope;GO:0005654//nucleoplasm |
| Alpha-1B-glycoprotein | gi\|21071030\|ref\|NP_570602.2\| | 5 | 11.7 | 58440 | 79 | 0.57 | - | - | GO:0005576//extracellular region |
| Apolipoprotein B variant (Fragment) | gi\|306569733\|gb\|ADN03360.1\| | 1 | 43.2 | 222441 | 9799 | 0.52 | GO:0010886//positive regulation of cholesterol storage;GO:0042953//lipoprotein transport;GO:0009615//response to virus;GO:0032496//response to lipopolysaccharide;GO:0045540//regulation of cholesterol biosynthetic process;GO:0010269//response to selenium ion;GO:0042159//lipoprotein catabolic process;GO:0034374//low-density lipoprotein particle remodeling;GO:0048844//artery morphogenesis;GO:0006898//receptor-mediated endocytosis;GO:0010744//positive regulation of macrophage derived foam cell differentiation;GO:0071356//cellular response to tumor necrosis factor;GO:0009566//fertilization;GO:0034383//low-density lipoprotein particle clearance;GO:0007283//spermatogenesis;GO:0009791//post-embryonic development;GO:0050900//leukocyte migration;GO:0034379//very-low-density lipoprotein particle assembly;GO:0019433//triglyceride catabolic process;GO:0030317//sperm motility;GO:0007596//blood coagulation;GO:0042158//lipoprotein biosynthetic process;GO:0030301//cholesterol transport;GO:0007399//nervous system development;GO:0009743//response to carbohydrate stimulus;GO:0071379//cellular response to prostaglandin stimulus;GO:0001701//in utero embryonic development;GO:0006642//triglyceride mobilization;GO:0042632//cholesterol homeostasis | GO:0050750//low-density lipoprotein particle receptor binding;GO:0005543//phospholipid binding;GO:0046982//protein heterodimerization activity;GO:0008201//heparin binding;GO:0019899//enzyme binding;GO:0017127//cholesterol transporter activity | GO:0030669//clathrin-coated endocytic vesicle membrane;GO:0034360//chylomicron remnant;GO:0005788//endoplasmic reticulum lumen;GO:0034361//very-low-density lipoprotein particle;GO:0005789//endoplasmic reticulum membrane;GO:0031904//endosome lumen;GO:0034363//intermediate-density lipoprotein particle;GO:0034359//mature chylomicron;GO:0005886//plasma membrane;GO:0034362//low-density lipoprotein particle;GO:0010008//endosome membrane;GO:0031983//vesicle lumen |
| Beta globin (Fragment) | gi\|193244921\|gb\|ACF16774.1\| | 3 | 39 | 14272 | 462 | 0.75 | GO:0042744//hydrogen peroxide catabolic process;GO:0051291//protein heterooligomerization;GO:0010942//positive regulation of cell death;GO:0015701//bicarbonate transport;GO:0008217//regulation of blood pressure;GO:0015671//oxygen transport;GO:0070293//renal absorption;GO:0030185//nitric oxide transport;GO:0045429//positive regulation of nitric oxide biosynthetic process;GO:0044281//small molecule metabolic process;GO:0050880//regulation of blood vessel size;GO:0007596//blood coagulation | GO:0004601//peroxidase activity;GO:0030492//hemoglobin binding;GO:0005344//oxygen transporter activity;GO:0019825//oxygen binding;GO:0031720//haptoglobin binding;GO:0020037//heme binding;GO:0005506//iron ion binding | GO:0031838//haptoglobin-hemoglobin complex;GO:0005833//hemoglobin complex |
| Calmodulin (Fragment) | gi\|74219094\|dbj\|BAE26689.1\| | 4 | 18.7 | 24209 | 60 | 0.41 | - | - |  |

^a^ According to the NCBI database.

^b^ Theoretical molecular mass.
